# PD55 Factors Associated With The Costs Of Inpatient Care For Preterm Born Infants In Brazil: A Nationwide Retrospective Study

**DOI:** 10.1017/S0266462325101876

**Published:** 2025-12-29

**Authors:** Aline Toledo, Julia Raffin, Emilie Batista Freire, Angela Ben, Johanna van Dongen, Esther Maas, Andre Santos, Rodrigo Luiz Carregaro, Judith Bosmans

## Abstract

**Introduction:**

Prematurity accounts for 75 percent of neonatal morbidity, imposing a substantial burden on healthcare services that extends beyond the neonatal period and leads to considerable healthcare costs. This study aimed to investigate factors associated with inpatient healthcare costs for preterm born infants in the first year of life in Brazil.

**Methods:**

A population-based cohort study was conducted from the perspective of the Brazilian public health system. We linked infants from the National Live Birth Information and Hospital Information Systems using deterministic record linkage. Infants born prematurely between January 2017 and December 2022 who were admitted to hospitals in the first year of life were included. Data on gestational age (GA), five-minute Apgar score (Apgar5), type of delivery, and inpatient healthcare costs were extracted from the databases. Generalized linear regression was used to analyze the association between GA, Apgar5, type of delivery, and the dependent variable of inpatient care costs.

**Results:**

A total of 196,375 preterm born infants were linked. Each additional week of GA was associated with a reduction in inpatient healthcare costs of BRL1,543.87 (USD285.90) (95% confidence interval [CI]: −BRL1568.85 [−USD290.52], −BRL1518.89 [−USD281.27]) and each additional point increase in Apgar5 score with a reduction of BRL722.67 (USD133.82) (95% CI: −BRL765.83 [−USD141.82], −BRL679.51 [−USD125.83]). Vaginal delivery was associated with a decrease of BRL1,350.26 (USD250.04) (95% CI: −BRL1429.51 [−USD264.72], −BRL1271.01 [−USD235.37]), compared with Cesarean section.

**Conclusions:**

Vaginal delivery and higher GA and Apgar5 score were associated with a significant reduction in inpatient care costs. Public policies aimed at reducing the rate of preterm births and promoting vaginal births in Brazil are essential for reducing hospital costs in the country.

